# *MALAT1* Long Non-Coding RNA: Functional Implications

**DOI:** 10.3390/ncrna6020022

**Published:** 2020-06-03

**Authors:** Gayatri Arun, Disha Aggarwal, David L. Spector

**Affiliations:** 1 Envisagenics, 101 Avenue of the Americas, New York, NY 10013, USA; garun@envisagenics.com; 2 Graduate Program in Genetics, Stony Brook University, Stony Brook, New York, NY 11794, USA; aggarwa@cshl.edu; 3 Cold Spring Harbor Laboratory, Cold Spring Harbor, New York, NY 11724, USA

**Keywords:** lncRNA, *MALAT1*, pre-mRNA splicing, non-coding RNA

## Abstract

The mammalian genome is pervasively transcribed and the functional significance of many long non-coding RNA (lncRNA) transcripts are gradually being elucidated. *Metastasis Associated Lung Adenocarcinoma Transcript 1* (*MALAT1*) is one of the most well-studied lncRNAs. *MALAT1* is a highly conserved nuclear retained lncRNA that is abundantly expressed in cells and tissues and has been shown to play a role in regulating genes at both the transcriptional and post-transcriptional levels in a context-dependent manner. However, *Malat1* has been shown to be dispensable for normal development and viability in mice. Interestingly, accumulating evidence suggests that *MALAT1* plays an important role in numerous diseases including cancer. Here, we discuss the current state-of-knowledge in regard to *MALAT1* with respect to its function, role in diseases, and the potential therapeutic opportunities for targeting *MALAT1* using antisense oligonucleotides and small molecules.

## 1. Introduction

The eukaryotic genome is transcribed into tens of thousands of non-coding RNAs adding additional complexity to the regulatory framework of cells and organisms. Long non-coding RNAs (lncRNAs) are emerging as a critical class of transcripts participating in a wide range of cellular functions. The current Gencode annotation estimates that there are at least 17,952 lncRNA genes (48,438 transcripts) in humans and 13,197 lncRNA genes (18,864 transcripts) in mice [1]. These genes are transcribed by RNA polymerase II and exhibit classical promoter and enhancer elements. The majority are capped, spliced, and polyadenylated, although some are single exon transcripts. Many lncRNAs undergo alternative pre-mRNA splicing and/or alternative cleavage and polyadenylation leading to multiple isoforms from the same locus [2,3]. While almost all eukaryotic species encode lncRNA genes, conservation of lncRNAs has been a topic of intense debate for over a decade [4,5,6]. Many lncRNAs show poor sequence level conservation, with some of them demonstrating structural conservation to some degree. In many instances, orthologs are identified by syntenic conservation followed by functional rescue experiments [7].

Despite the large number of annotated lncRNAs, a large number (>80%) are expressed at very low levels, few transcripts per cell, and the majority of these transcripts are restricted to just one or a few cell types and/or specific developmental stages or physiologic conditions [8,9]. In addition to cell type specific expression, many lncRNAs also display a specific sub-cellular localization being restricted to cytoplasmic or nuclear compartments or association with specific organelles, such as polycomb bodies, stress granules, nuclear speckles, paraspeckles, etc. [10]. More recent evidence suggests that lncRNAs actively contribute to phase separation in large ribonucleoprotein complexes giving rise to membrane-less organelles inside the cell [11,12,13,14]. Given the remarkable diversity of this class of RNAs, it is reasonable to envision that they contribute in a structural, functional, and/or regulatory capacity in a wide range of cellular/nuclear processes [15]. *Metastasis Associated Lung Adenocarcinoma Transcript 1* (*MALAT1*) is one of the most widely studied nuclear restricted lncRNAs that has gained much attention in recent years due to its abundance, rather ubiquitous expression, and apparent role in various disease manifestations. Here, we discuss the current state-of-knowledge in regard to *MALAT1* function and its putative role in several disease states, including cancer. 

## 2. *MALAT1*–Basic Characteristics

*MALAT1*, also known as *Nuclear Enriched Abundant Transcript* 2 (*NEAT2*) [16] was first identified in a microarray screen of tumors from patients with non-small cell lung cancer, and was found to be upregulated in the tumors with a higher propensity to metastasize [17]. The *MALAT1* gene is encoded on human chromosome 11q13.1 and mouse chromosome 19qA. It is located in a gene dense region with a very high syntenic evolutionary conservation [8]. *MALAT1* exhibits a remarkable sequence conservation with greater than 50% overall conservation in vertebrates and greater than 80% conservation at the 3′ end of the transcript [18,19,20]. This is one of the key distinguishing features of *MALAT1* as very few lncRNAs show such a high level of evolutionary conservation. Less than 10% of all lncRNAs show exonic as well as structural conservation equivalent to that of protein coding genes [21,22]. The *MALAT1* transcript has been confirmed as a non-coding RNA as it exhibits low protein-coding potential using two independent coding potential calculating algorithms CPC2 [23] and CPAT [24]. 

Human *MALAT1* is ~8.7 knt long, whereas the mouse RNA is 6.7 knt long [8]. It is transcribed by RNA polymerase II and its promoter has an accessible open chromatin architecture, which has been shown in several high-throughput studies and DNAse sensitivity assays [8]. The steady state expression level of *MALAT1* is very high and is comparable to highly transcribed housekeeping genes, such as β-*Actin* [8]. Further, *MALAT1* is ubiquitously expressed across all tissues with an average median expression of about 150 TPM (transcripts per million) with highest expression in ovaries with a median expression of 287 TPM [25]. The abundance of *MALAT1* in cells is likely the consequence of strong promoter activity coupled with increased stability of the transcribed RNA [26,27]. *MALAT1* was originally classified as an intron-less transcript with a genomically-encoded poly A tract. However, with a number of deep sequencing efforts, several alternatively spliced isoforms and transcripts with alternative transcription start sites have been identified that are expressed during different physiological states such as cancer [8,28]. In addition, a natural antisense transcript *TALAM1* has also been identified and has been suggested to play a role in a feed-forward positive regulatory loop to maintain the high cellular levels of *MALAT1* and contribute to its stability [29]. Interestingly, simultaneous knockdown of both *MALAT1* and *TALAM1* in breast cancer cells led to a stronger, synergistic decrease in migration and invasion of these cells and reduced metastasis to the lungs in a mouse model [30].

The *MALAT1* primary transcript is processed to yield the well characterized nuclear retained *MALAT1* transcript, and from its 3′ end a tRNA-like small RNA [27]. The biogenesis of the small RNA is mediated by the tRNA processing machinery, RNase P and RNase Z. The 61-nucleotide tRNA- like *MALAT1*-associated small cytoplasmic RNA (mascRNA) is exported to the cytoplasm [27]. The resultant 3′ end of the nuclear *MALAT1* transcript post-processing is not polyadenylated, however, it contains a genomically-encoded poly(A)-rich stretch which pairs with an upstream U-rich region and then adopts a unique triple helical confirmation [31,32,33,34]. This triple helical structure was first identified in the PAN (polyadenylated nuclear) RNA produced by the human oncogenic Kaposi sarcoma-associated γ-herpesvirus (KSHV) *PAN* RNA [35,36]. The only other human or mouse RNA that exhibits such a structure is the ~20 knt Men-β (*NEAT1_2*) RNA [33,34]. The triple helical structure has been shown to confer stability and nuclear localization to *MALAT1* in the absence of a true poly(A) tail and has been shown to bind several RNA binding proteins (RBPs) including METTL16 which is an m^6^A RNA methyl-transferase [33,37]. Specific triplex-disrupting mutations lead to *MALAT1* degradation and loss of nuclear accumulation [38]. Further, a class of RNAs containing a similar 3′ end including a triple helix structure have been identified in several other species including lizards, where they have been shown to play a role in piRNA biogenesis in testicular cells [32]. Taken together, the *MALAT1* locus displays remarkable evolutionarily conserved secondary and tertiary structural features and an unusual 3′ end processing mechanism. It is not fully apparent whether the full length *MALAT1* RNA with its 3′ end triplex structure, the processed tRNA-like RNA, and the natural antisense RNA have a concerted mechanism of action or if each component derived from this interesting locus has a disparate function. Further, high-throughput chemical mapping experiments have highlighted extensive epi-transciptomic changes in the *MALAT1* transcript, for example, m^6^A, pseudouridylation and 5-methyl cytosine [39,40,41]. It has been shown that the addition of m^6^A at the A2577 position could destabilize the hairpin stem of *MALAT1*, making it accessible for RNA-binding proteins such as HNRNPC [42]. Additional detailed molecular studies elucidating the transcriptional and post-transcriptional regulation of the *MALAT1* locus will address these issues and allow us to further understand the regulation and function of the *MALAT1* locus.

## 3. *MALAT1* Localization

**Figure 1:**
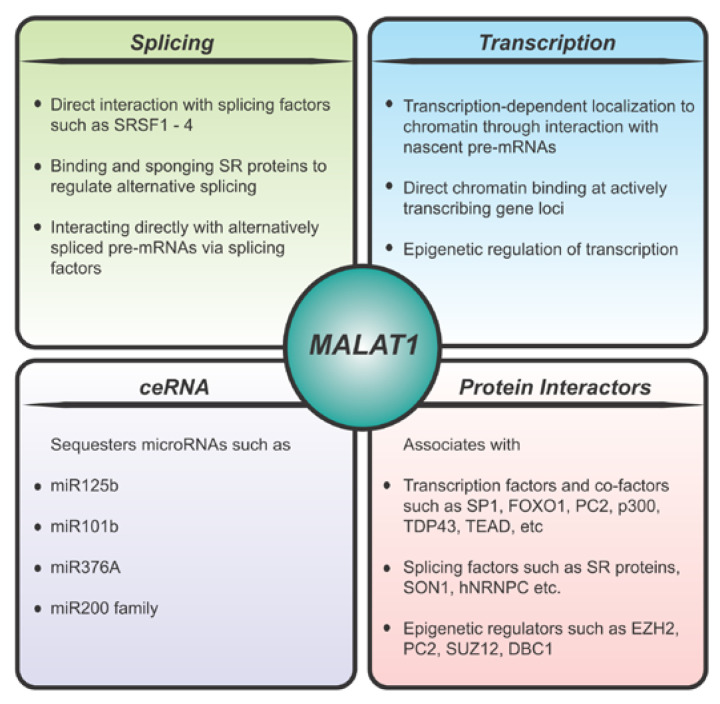
Molecular mechanisms of *MALAT1* function.

*MALAT1* is a nuclear retained RNA that localizes to nuclear domains known as nuclear speckles [16]. Nuclear speckles are enriched in pre-mRNA processing factors, as well as some transcription factors, and play a critical role in coordinating transcriptional and post-transcriptional gene regulation [43]. *MALAT1* has been shown to be enriched at the periphery of the nuclear speckles with pre-mRNA splicing factors localized more internally [44]. The previously described triple-helix element and other *MALAT1* domains have been shown to facilitate the nuclear retention and localization of *MALAT1* [31,45]. Although *Malat1* localizes to nuclear speckles it is not required for the formation of nuclear speckles [16] and knockout of *Malat1* has no overall effect on the assembly, number, size, distribution, or maintenance of nuclear speckles [46]. As such *Malat1* is not a candidate to play a role in regulating the process of phase separation in the formation of nuclear speckles. Several nuclear speckle components such as, RNPS1, SRm160, and IBP160, were found to be essential factors for the localization of *MALAT1* to nuclear speckles, and the proper localization was found to be mediated by two distinct regions of *MALAT1* (1777–3600 nt and 5185–6982 nt) [45,47]. In addition, *Malat1* has been shown to bind to several other pre-mRNA splicing factors that are enriched in nuclear speckles such as SRSF1, SON1, hnRNPC, hnRNPH1, etc. (Figure 1) [47,48,49]. *MALAT1* has also been shown to modulate recruitment of splicing factors to actively transcribing loci in human cell lines [26,47,50] thereby regulating alternative pre-mRNA splicing of a number of pre-mRNAs (Figure 1). Further, CRISPR screening studies, identified both positive (DHX15, DDX4,2, hnRNPH1 and hnRNPK) and negative (hnRNPA1, hnRNPL, and PCBP1) regulators of the nuclear speckle localization of *MALAT1*. It was suggested that negative regulators could compete with the factors that recruit *MALAT1* to nuclear speckles thereby dissociating *MALAT1* from nuclear speckles upon transcriptional inhibition suggesting a role for *MALAT1* in transcriptional regulation [51]. In addition to the above mentioned components contributing to the localization of *Malat1*, recently it has been shown that a SINE element in the *Malat1* 5′ end associates with HNRNPK, KHDRBS1, and TRA2A contributing to its nuclear localization [52]. A *MALAT1* SINE deletion mutant localizes diffusely in the nucleus and is frequently transported to the cytoplasm resulting in the formation of cytotoxic insoluble TDP-43 inclusions in both the cytoplasm and nucleus [52].

In addition to its nuclear speckle localization, *MALAT1* has also been shown to be associated with chromatin. High-throughput chromatin-RNA binding assays such as CHART and ChIRP [53,54] have identified *MALAT1* as a highly enriched RNA in the chromatin fraction, where it has been shown to be associated with transcriptionally active genes. In another study, *MALAT1* was shown to coordinate the relocation of genes from polycomb bodies to transcriptionally active sites in a serum responsive manner. This mechanism was directed through binding to several members of the polycomb group proteins such as PC2, EZH2, and SUZ12, thereby regulating the transcriptional status of a number of PRC2 target genes by relieving their repression (Figure 1) [55,56,57]. Further, it has been shown that *MALAT1* can target CTCF binding sites and active promoters [58]. Using CHIA-PET technology, it was shown that 3D genome organization impacts *MALAT1* binding to target genes and *MALAT1* binding sites were involved in both CTCF- and RNAPII-mediated chromatin interactions [58]. Specifically, such a long-range interaction was shown on the LTBP3 promoter which has been previously shown to be regulated by *MALAT1* [59]. Additional high-throughput experiments, such as MARGI and GRID-seq, have also revealed extensive binding of *MALAT1* to thousands of genomic loci in a cell-type specific manner [60,61].

## 4. Molecular Function of *MALAT1*

**Figure 2:**
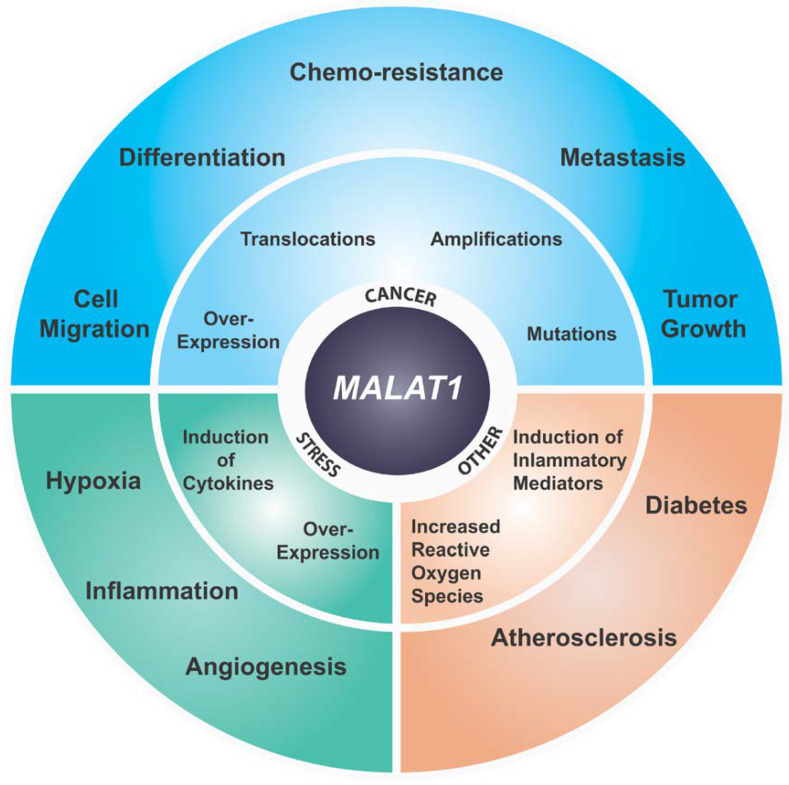
Summary of known disease implications of *MALAT1*.

Numerous mechanisms of action have been proposed to explain the role of *MALAT1* (Figure 1) in a wide range of physiological states (Figure 2). A significant number of studies have supported a function for *MALAT1* based on its defined subnuclear localization and have proposed that *MALAT1* either plays a role in transcription, directly or indirectly, and/or regulates alternative pre-mRNA splicing [62]. The splicing role for *MALAT1* is directly related to its localization in nuclear speckles, a sub-nuclear body enriched in pre-mRNA splicing factors [16,26,47]. A number of studies have demonstrated altered pre-mRNA splicing upon *MALAT1* knockdown in cells [47,63,64]. In addition, *MALAT1* has been shown to regulate the phosphorylation status of an SR splicing factor thereby regulating its speckle localization and its role in alternative pre-mRNA splicing [47]. Other studies have shown that *MALAT1* may directly participate in pre-mRNA splicing of actively transcribed genes by recruiting splicing factors to the pre-mRNA [65]. In addition to those factors described in the previous section, *MALAT1* has been shown to bind to several SR proteins such as SRSF1, SRSF3, SRSF2, and other RBPs, such as HNRNPL and TDP-43 [47,49,64,66,67]. Taken together these studies indicate a role for *MALAT1* directly or indirectly in the regulation of pre-mRNA splicing.

A number of studies have also shown a role for *MALAT1* at the transcriptional level (Figure 1). For example, in vivo cross-linking studies have shown *MALAT1* binding to chromatin of actively transcribing genes and regulating their expression at the transcriptional level [53]. In addition, *MALAT1* also binds to a number of transcription factors and transcriptional co-activators, such as LTBP3, FOXO1, PC2, HMGA2, etc. [55,59,68,69]. Using RNA reverse transcription-associated trap sequencing (RAT-seq), *MALAT1* was shown to increase proliferation and migration of breast cancer cells via binding to the *EEF1A1* promoter and upregulating its expression epigenetically [70]. *MALAT1* has also been shown to epigenetically upregulate transcriptional activators of proteosome subunit genes in multiple myeloma cells [71]. *MALAT1* has been shown to bind DBC1 causing deacetylation of p53, thus promoting cell proliferation and inhibiting cell apoptosis [49]. Overexpression or knockdown of *MALAT1* in mammalian cells under a wide range of physiological conditions influences transcriptional changes in a context specific manner [63,70,72,73,74,75]. Based on multiple lines of evidence *MALAT1* may likely influence both transcription as well as pre-mRNA splicing (Figure 1). With more recent studies demonstrating transcription-coupled splicing in determining alternative splice-site choice and alternative polyadenylation [76,77], it is tempting to speculate that *MALAT1* may directly coordinate these events in a context specific manner by either scaffolding the protein complexes or acting as a chaperone targeting the transcription/pre-mRNA splicing machinery to the appropriate genes.

In addition to influencing splicing and transcription, *MALAT1* has been shown to act as a competing endogenous RNA (ceRNA) or miRNA sponge to sequester miRNAs under various conditions (Figure 1). For example, it has been shown that miR-125b can bind to *MALAT1* to downregulate its expression and inhibit bladder cancer development [78]. It has also been reported that *MALAT1* regulates Rac1 expression by acting as a ceRNA for miR-101b in liver fibrosis [79]. *MALAT1* has also been shown to promote development of osteosarcoma by targeting TGFA via miR-376A [80]. Further, *MALAT1* has been shown to induce EMT during endometriosis [81] and metastasis in clear cell kidney carcinoma mouse models [82] via the miR200/Zeb axis by acting as a sponge for the miR200 family. While these studies allude to a role of *MALAT1* in sequestering miRNAs, it is not clear if this is attributed to the nuclear function of *MALAT1* as the majority of these miRNAs are enriched in the cytoplasmic compartment. Perhaps *MALAT1* sequesters miRNAs in the early nuclear stage of pre-miRNA processing as *MALAT1* does not appear to shuttle between the nuclear and cytoplasmic compartments. For a better understanding of these processes, the context of *MALAT1* function and localization needs to be more thoroughly investigated.

## 5. *MALAT1* is Dispensable for Normal Physiology

While it is compelling to speculate that *MALAT1* plays a very critical cellular function, three independent knockout (KO) mouse models generated by different groups concluded that loss of *Malat1* did not have an impact on normal mouse physiology or development [46,83,84]. In these mouse KO models generated using different strategies, *Malat1* loss did not affect the normal development of the mice and adult mice did not exhibit any aberrant phenotypes. Zhang et al. have reported a 1.5–2-fold upregulation of several genes neighboring *Malat1* in brain tissues upon KO of a 3.5 kb region surrounding the *Malat1* promoter suggesting a *cis* acting role for *Malat1*. However, this upregulation was not observed in other tissues, including mammary tissue (unpublished data). A second group reported a modest down-regulation of the *Neat1* transcript in intestines of *Malat1* KO mice [84]. A third group reported KO of *Malat1* did not affect proliferation or cell cycle progression in human lung or liver cancer cells. In addition, a KO *Malat1* mouse did not result in any obvious phenotype or histological abnormality [83]. One logical explanation of these findings is that there may be functional redundancy for the *Malat1* RNA under normal physiologic conditions as is the case for many critical genes [85,86].

## 6. *MALAT1* and Cancer

*MALAT1* was initially identified as an RNA whose expression is elevated in primary lung tumor that had a higher propensity to metastasize [17]. Since this initial study overexpression of this lncRNA has been reported in over 20 different solid or lymphoid tumors specifically correlating its higher expression to tumor progression and metastasis (Figure 2) [17,63,71,87,88,89,90,91,92,93,94,95,96,97,98,99]. Depending on the type and stage of cancer, the relative upregulation was found be from 1.5–10 fold [17,63,100,101]. Higher expression of *MALAT1* has been shown to be associated with poor prognosis in a variety of solid cancers and hematopoietic cancers [102,103,104,105]. Additionally, *MALAT1* overexpression has been associated with metastasis in lung, breast and liver cancers [17,63,64,102]. *Malat1* loss or knockdown in a murine metastatic cancer model resulted in differentiation of primary tumors and a significant reduction in metastasis [63]. Additionally, both *Malat1* knockdown and genetic KO in a lung cancer homing model reduced homing to the lungs of lung cancer cells [101]. Similar observations have been reported in CRC, esophageal carcinoma, gallbladder, cervical cancer, and prostate cancer where knockdown of *MALAT1* abrogated tumor growth and/or metastasis in the respective cell line-derived models and/or PDX mouse models [69,87,106,107,108,109]. In many of these studies *MALAT1* knockdown affected transcription and/or pre-mRNA splicing of critical genes involved in migration and cell adhesion in addition to genes involved in critical cancer pathways. Additionally, overexpression of the 5′ *Malat1* fragment was found to be sufficient to transform mouse primary embryonic fibroblast cells resulting in increased colony formation in soft a gar assays [110]. Interestingly, Gao et al. have demonstrated that expression of the *Malat1* 5′ region can induce metastasis in the non-metastatic 4T07 murine mammary cancer cell line suggesting a gain of function for the *Malat1* 5′ fragment in promoting metastasis [111]. In addition, *MALAT1* overexpression has been shown to be associated with drug resistance in breast cancer, CRC, prostate cancer, etc. (Figure 2) [112,113,114]. Despite accumulating evidence that *MALAT1* plays a pro-oncogenic and pro-metastatic role in a wide range of cancers, including mammary cancer, as discussed above, a few recent studies reported a tumor suppressor-like role for *Malat1* [74,115,116]. The significance of these later findings is unclear as they are contradictory to a large body of data supporting a pro-oncogenic role for *MALAT1*. Additional studies are necessary to clarify these differences [117].

*MALAT1* is a highly-conserved noncoding RNA gene transcribed from the human 11q13 locus which has been shown to exhibit copy number changes, translocations, or mutations in several cancer types. *MALAT1* translocation to *TFEB* has been reported in renal cell carcinoma [118]. Translocation of the 5′ region of *MALAT1* to *Gli1* has been found to be oncogenic in an aggressive form of Gastroblastoma [119]. Further, the *MALAT1* locus was shown to exhibit tandem duplication in some breast cancers resulting in increased dosage of the gene [120]. Apart from chromosomal aberrations in the *MALAT1* locus in cancer, WGS studies from patient tumors have found that *MALAT1* is a frequently mutated gene in breast and other cancer types [121,122]. A number of hotspot mutations have been identified in the *MALAT1* gene that are mostly clustered in the 3 kb–4.3 kb region, although the role of such short indels and point mutations in this gene is unclear [122]. Recent PCWGA suggests that *MALAT1* mutations may be a consequence of the high level of transcription associated with the gene and an inherently fragile genomic locus, and may not necessarily represent driver mutations [123,124]. However, additional studies are warranted to assess whether these aberrations may interfere with the above functions of *MALAT1* or represent mutations that promote tumorigenesis.

Finally, meta-analysis of transcriptomic datasets has also shown *MALAT1* to be upregulated in several cancer tissues such as lung, CRC, prostate, breast, etc. cancer compared to normal tissues [125]. Analysis of TCGA data from breast, lung, prostate, and glioma cancers have identified overexpression of *MALAT1* associated with poor prognosis and reduced metastasis-free survival [102,103,104,105]. Higher levels of *MALAT1* have also been observed in circulating RNAs, and also RNAs extracted from exosomes from cancer patients [126,127,128]. *MALAT1* levels in urine and urinary exosomes have been evaluated in prostate and bladder cancer respectively for developing *MALAT1* as a non-invasive prognostic biomarker [129,130]. It is compelling that this wide range of studies have identified *MALAT1* as being strongly enriched in various body fluids of cancer patients and warrants *MALAT1* to be further evaluated as a potential prognostic or diagnostic marker. However, the specificity of such a diagnostic assay will be challenging, as *MALAT1* is also an abundant RNA in most normal tissues, which can contribute to significant noise in such analysis.

## 7. *MALAT1* and Stress Responses

While no apparent phenotype has been observed upon *Malat1* loss in knockout mice, differential expression of *MALAT1* has been reported under various physiological stresses such as serum starvation, hypoxia etc. [55,64,131]. Additionally, it has been shown that *MALAT1* enhances glycolysis, and inhibits gluconeogenesis, via elevated translation of the transcription factor TCF7L2 and as such also plays a role in metabolic stress [132]. Knockout of other important genes, such as RPL, Cyclin D, etc., show a lack of phenotype under normal conditions due to functional redundancy, whereas upon physiological stress they manifest a phenotype [85,86]. Consistent with this hypothesis, *Malat1* KO mice crossed with breast tumor bearing models display a tumor differentiation phenotype [63]. In addition, *MALAT1* localization and function has been shown to be altered during serum starvation [55]. *Malat1* was also demonstrated to be induced in kidneys of hypoxic mice [133], and *Malat1* was identified as one of the most upregulated non-coding transcripts upon hypoxia in a breast cancer cell line [134]. *MALAT1* has been shown to be regulated by HIF1α, a key transcription factor during the hypoxic response [134,135]. *MALAT1* knockdown also influences the expression of proangiogenic isoforms of VEGFa which is a classic HIF1α regulated gene [73]. In multiple myeloma, *MALAT1* was shown to be a target of KDM3A, whose upregulation resulted in accumulation of HIF-1α, and induction of glycolytic genes under hypoxia conditions [136]. An additional study has demonstrated that cancer cell-specific chromatin-chromatin interactions are formed at the *MALAT1* locus under hypoxic stress, thereby implicating a novel role of *MALAT1* in regulating hypoxic response in cancer [131]. Collectively, these studies indicate a direct role for *MALAT1* in hypoxic stress which is responsible for significant pathological consequences in cancer including angiogenesis and metastasis.

*MALAT1* has also been shown to play a critical role in regulating the A-NHEJ pathway during B cell class switch recombination [137]. Further, several studies have identified *MALAT1* as a regulator of TRP53 [75,138] and knockdown of *MALAT1* was shown to result in increased H2Ax foci [75] suggesting that *MALAT1* plays a more general role in the double-strand break response and genotoxic stress. Chemotherapeutic agents are known to cause genotoxic stress and, interestingly, *MALAT1* was significantly upregulated by chemotherapeutic agents in extramedullary myeloma suggesting that it could be a stress responsive gene [139]. *MALAT1* has also been shown to be a target for chemo-sensitization of GBM wherein it is regulated by members of the TP53 family [104,140]. Similar observations of upregulation of *MALAT1* has been reported in drug resistance phenotypes in lung, prostate and other cancers [112,141,142,143]. This is a rather intriguing observation as many studies mentioned above have observed a strong correlation between *MALAT1* expression and the development of chemo-resistance in cancer. Further investigations along this line are warranted in order to understand the role of *MALAT1* in the development of the drug resistance phenotype in cancers and to identify potential combinatorial therapeutic opportunities to target *MALAT1* to augment chemotherapeutic response.

## 8. *MALAT1* in Other Diseases

In addition to cancer, studies have identified *MALAT1* upregulation in a wide range of other pathological indications as summarized in Figure 2 [144]. A significant number of studies have directly implicated *MALAT1* in development of diabetes and insulin signaling. An early study identified *MALAT1* upregulation in endothelial cells subjected to high glucose treatment [145]. *MALAT1* was also found to play an important role in regulating insulin sensitivity by regulating NRF2 activity and suppressing JNK signaling with concomitant insulin-induced phosphorylation of Akt [146]. Additionally, a novel signaling nexus involving *MALAT1* and SAA3 has been identified which turns on inflammatory mediators in the endothelium in response to glucose level suggesting a role for *MALAT1* in micro- and macro-vascular complications of diabetes [145]. More recently, several studies have identified dysregulation of *MALAT1* expression in multiple pathophysiological complications of diabetes including retinopathy, artherosclerosis, cerebrovascular disorder, renal disorders, etc. [144]. Further, molecular studies of several of these pathological indications have converged upon identifying a deregulated inflammatory response induced by altered *MALAT1* level. For example, a number of inflammatory molecules such as TNFα and IL6 have been shown to be increased in *MALAT1* upregulated cells [147]. Additionally, shRNA mediated the knockdown of *MALAT1* ameliorated the inflammatory injury after lung transplant ischemia-reperfusion by inhibiting chemotaxis of neutrophils through p300-mediated downregulation of IL-8 [69]. Further, using *Malat1* KO mice it was demonstrated that reduced levels of *Malat1* augment atherosclerotic lesion formation in mice and are associated with human atherosclerotic disease [148]. They also showed that pro-atherosclerotic effects observed in *Malat1*^-/-^ mice were mainly caused by enhanced accumulation of hematopoietic cells involved in inflammatory response [148].

## 9. Therapeutic Targeting of *MALAT1*

Given the diverse role of *MALAT1* in cancer and other disease areas such as diabetes and inflammation, *MALAT1* is being actively investigated as a potential therapeutic target using different modalities. Pre-clinical studies using breast and lung cancer models targeting *Malat1* using antisense Gapmer oligonucleotides have resulted in an anti-tumor and anti-metastatic outcome in both studies [63,101]. Gapmer oligonucleotides are short single-stranded RNA-DNA-RNA hybrids that bind to complementary RNA sequences and cause degradation by invoking an RNaseH response [149]. Gapmers are emerging as a promising approach to target multiple lncRNAs [62]. In addition, targeting the *Malat1* and AR-v7 axis using *Malat1*-short interfering RNAs (siRNAs) in enzalutamide-resistant prostate cancer cell lines and mouse models suppressed enzalutamide-resistant prostate cancer progression [113]. Similar studies using *Malat1*-targeting siRNAs have been conducted in other cancer types, such as glioblastoma, ovarian, colorectal (CRC), gallbladder, gastric, osteosarcoma, and esophageal, etc. [88,106,107,140,142,150,151,152]. *MALAT1* gapmer oligonucleotides conjugated to single- walled carbon nanotubes delivered systemically into mice resulted in significant inhibition of multiple myeloma growth [153]. Further, small molecules specifically targeting the *MALAT1* triple helix structure have been identified and they lay the foundation for new classes of anticancer therapeutics for the treatment and investigation of *MALAT1*-driven cancers [154]. Together, these studies provide compelling evidence for targeting *MALAT1* in multiple cancer types to achieve a therapeutic benefit. Given that *Malat1* knockout mice are healthy and fertile, *MALAT1* targeting in cancer can be a potentially viable mechanism to evade the emergence of a drug resistant phenotype in *MALAT1* elevated chemo-resistant cancers or to achieve a significant anti-tumor and anti-metastatic effect in *MALAT1* overexpressing cancers without causing any adverse side effects to healthy tissues. Based upon the significant body of pre-clinical data *MALAT1* is poised to be targeted by antisense or small molecule drugs to impact cancer progression and other inflammatory and metabolic disease indications.

## 10. Summary and Conclusions

*MALAT1* breaks all of the “rules” when it comes to a lncRNA: it is highly abundant, well-conserved, is expressed broadly among different cell types and tissues, and exhibits an unusual 3′-end processing mechanism. As discussed in this review *MALAT1* appears to function in a context-dependent manner and as such has been implicated in a wide array of functions. Its expression level has been shown to be altered in many different physiologic states including being upregulated in a plethora of different cancer types, as well as exhibiting altered expression in many other diseases. One intriguing hypothesis derived from these studies is that *MALAT1* functions in a context-dependent manner, at the level of pathways rather than individual gene(s), and as such may represent an outstanding therapeutic target as it may impact multiple nodes of particular pathways thereby minimizing the drug resistance problem in cancer treatment. Future studies will certainly add more to the intriguing basic biology of *MALAT1* and bring it closer to having clinical impact.
